# Propofol Protects Rat Cardiomyocytes from Anthracycline-Induced Apoptosis by Regulating MicroRNA-181a *In Vitro* and *In Vivo*

**DOI:** 10.1155/2018/2109216

**Published:** 2018-04-18

**Authors:** Hongwei Zhao, Xiaobei Zhang, Ying Zheng, Yuan Li, Xiaokun Wang, Nan Hu, Peng Zhou, Kaiyuan Wang

**Affiliations:** ^1^Department of Anesthesiology, Tianjin Medical University Cancer Institute and Hospital, National Clinical Research Center for Cancer, Tianjin 300060, China; ^2^Key Laboratory of Cancer Prevention and Therapy, Tianjin 300060, China; ^3^Tianjin's Clinical Research Center for Cancer, Tianjin 300060, China

## Abstract

We aimed to evaluate the cardioprotective effect and mechanism of propofol in anthracycline-induced cardiomyocyte apoptosis. We selected the rat myocardial cell line, H9c2, and primary cardiomyocytes for *in vitro* study. The cardiomyocytes were treated with vehicle, Adriamycin® (ADM), propofol, or a combination of ADM and propofol. The proportion of apoptotic cells and the expression of miR-181a were detected by flow cytometry and real-time PCR, respectively. Luciferase assays were performed to explore the direct target gene of miR-181a. *In vivo* assay, rats were randomly divided into different treatment groups. The apoptosis index was determined by TUNEL staining, and the expression of miR-181a and STAT3 in heart tissue was detected. The antiproliferative effect of ADM alone was significantly greater than that of ADM plus propofol. A significantly greater decrease in the proportion of apoptotic cells and in miR-181a expression was observed in the combination treatment group compared with that in the ADM groups *in vitro* and *in vivo*. The loss-of-function of miR-181a in H9c2 of ADM treatment resulted in increased Bcl-2 and decreased Bax. MiR-181a suppressed Bcl-2 expression through direct targeting of the Bcl-2 transcript. Propofol reduced anthracycline-induced apoptosis in cardiomyocytes via targeting miR-181a/Bcl-2, and a negative correlation between miR-181a and Bcl-2 was observed.

## 1. Introduction

The use of anthracycline (ANT) for the chemotherapy of various malignancies has been hampered by their cardiac toxicity. The most severe is the chronic forms of cardiotoxicity, characterized by irreversible cardiac damage and congestive heart failure, which remain a major problem 50 years after the discovery of daunorubicin and doxorubicin (DOX), also known as Adriamycin (ADM), and their introduction in clinics [1]. The pathophysiology of ANT-induced cardiotoxicity is still not fully understood; it remains a subject of debate and considerable controversy. Recently, several scientists suggested that ANT-induced cardiotoxicity and myocardial dysfunction were associated with cardiomyocyte apoptosis [2–4]. Our previous study indicated that anthracycline could induce the apoptosis of cardiomyocytes in rats. Therefore, we focused on the interpretation of the mechanism of anthracycline-induced apoptosis in cardiomyocytes in the presence or absence of propofol.

Recent studies have demonstrated that microRNAs, as endogenous regulators of gene expression, were involved in the regulation of cardiovascular disease in several biological processes, including endothelial cell dysfunction, cell adhesion, and cardiomyocyte proliferation and apoptosis [5–7]. The importance of miR-181a in cardiomyocyte apoptosis has been confirmed in numerous experiments [8, 9]; however, the role of miR-181a in anthracycline-induced myocardial injury requires further study.

Propofol (2,6-diisopropylphenol), known as “milk of anesthesia,” is one of the most popular intravenous anesthetic agents in modern medicine. It is commonly used for the induction and maintenance of anesthesia and procedural and critical care sedation in children [10]. The cardioprotective activities of propofol were shown by in the protection of myocardial cells from ischemia-reperfusion injury (IRI) *in vitro* [11, 12], *in vivo* [13], and in clinical trials [14, 15]. Only a few studies have investigated the cardioprotective effect of propofol in ANT-induced cardiotoxicity [16]. In this study, we aimed to evaluate the protective effect and mechanism of propofol in the regulation of ANT-induced apoptosis in cardiomyocytes *in vitro* and *in vivo* and the role of miR-181a in the regulation of ANT-induced cardiomyocyte apoptosis.

## 2. Material and Methods

### 2.1. Material

#### 2.1.1. Drugs and Chemicals

Doxorubicin (Adriamycin) was provided by Haimen Pharmaceutical (Zhejiang, China). Propofol was provided as a clinical formulation by the Pharmacy of Tianjin Medical University Cancer Institute and Hospital. Doxorubicin was diluted with sterile water to a final concentration of 100 mM and stored at 4°C. Propofol was diluted with DMSO to a final concentration of 10 mg/ml and stored at 4°C.

#### 2.1.2. Objects

The rat myocardial cell line H9c2 was used from our laboratory, cultured in DMEM supplemented with 10% FBS and penicillin/streptomycin double-antibiotic at 37°C in a 5% CO_2_ incubator.

Sprague–Dawley and F344 rats, between 6 and 9 weeks old, were purchased from Laboratory Animal Science Department of the Peking University Health Science Center (license number SCXK (Jing) 2006–2008). The animals were maintained in a naturally ventilated room, with a temperature of 25–28°C, relative humidity of 70%–85%, and a 12 h light/dark cycle. The animals were provided with standardized laboratory rat pellet food and water ad libitum and weighed weekly.

#### 2.1.3. Experimental Instruments

The flow cytometer (BD FACS Aria I), ELISA reader, incubator, centrifuges, and other experimental instruments were provided by the Core Laboratory of the Tianjin Medical University Cancer Institute and Hospital.

### 2.2. Methods

#### 2.2.1. Primary Cardiomyocyte Isolation and Culture

Twenty Sprague–Dawley (SD) rats were used for the isolation and culture of primary cardiomyocytes. After whole hearts were extracted aseptically, the pericardium and atrium were removed and washed several times with ice-cold PBS to remove residual blood and debris. The residual ventricular parts of the hearts were minced into 1-2 mm^3^ tissue blocks under sterile conditions. The tissues were then repeatedly digested with freshly prepared compound digestive enzyme [collagenase II (Gibco) 1 mg/ml, hyaluronidase (Gibco), 0.2 mg/ml in Hank's solution (Gibco), sterile filtered, and pH 7.2] for 20 min in a 37°C incubator until the tissue blocks were completely digested and then filtered through a 200 *μ*m cell strainer. Filtered cardiac cells were cultured in a flask and incubated at 37°C for 1 h, whereas fibroblasts were mostly adherent, the cell supernatant enriched with ventricular myocytes was gently aspirated and the suspensions were centrifuged at 800 rpm for 5 min at 4°C to remove debris. The cell cultures were resuspended in cold DMEM/F12 medium (Gibco) supplemented with 10% FBS (Gibco) and penicillin and streptomycin (Gibco).

#### 2.2.2. MTT Assay

The MTT (3-(4-5-dimethylthiazol-2-yl)-2, 5-diphenyl tetrazolium bromide dye) reduction assay was performed to compare the effects of propofol, ADM, and the combination treatment. Each condition was replicated in five wells. The inhibitory concentration of propofol in the combination treatment was maintained at 1 *μ*g/ml, and the concentrations of ADM were 100 nM, 500 nM, 1 *μ*M, and 10 *μ*M.

Twenty-four hours after treatment, 20 *μ*l MTT (5 mg/ml in PBS) was added to each well. After 4 h, the supernatant was discarded, and 150 *μ*l dimethyl sulfoxide (DMSO) was added to each well, and the plate was vortex mixed for 10 min. The OD (optical density) of each well was determined by using an ELISA reader, and the drug action curve was plotted.

#### 2.2.3. Detection of Apoptosis by Flow Cytometry

H9c2 cells and primary cardiomyocytes were placed in 6-well plates. The cells were divided into four groups: (1) control group: blank control; (2) prop group: 1 *μ*g/ml propofol; (3) ADM group: 10 *μ*M Adriamycin; and (4) prop + ADM group: 1 *μ*g/ml propofol + 10 *μ*M Adriamycin. Culture medium, containing 0.5% DMSO, was added to the control group. After treatment, the cells were cultured for 24 h at 37°C in a 5% CO_2_ incubator prior to collection. To detect apoptosis, the cells were collected as a single cell solution, and annexin-FITC and propidium iodide (PI) were added to the cells. The cells were mixed thoroughly with the reagents and incubated at 18–21°C (room temperature) in the dark for 5–15 min. Within 1 h, flow cytometry was used for the detection of apoptosis to evaluate the effects of the drugs.

#### 2.2.4. Quantitative Real-Time PCR

After the treatments were applied to the cells, total RNA was extracted from H9c2 cells and primary cardiomyocytes by using the Trizol Reagent (Invitrogen). The RT primers sequence of miR-181a was 5′-GTCGT ATCCA GTGCG TGTCG TGGAG TCGGC AATTG CACTG GATAC GACAC TCAC-3-′. The SYBR green stem-loop RT-PCR method was used to assess the expression levels of the miRNAs. The PCR conditions were as follows: 95°C for 10 min, followed by 40 cycles of 95°C for 15 s, and 60°C for 1 min. Each sample was analyzed in duplicate with RNA preparations from three independent experiments. The analysis was performed by using the ABI PRISM 7900 system (Applied Biosystems). The fold change in the expression of each gene was calculated by using the 2^ΔΔCT^ method, with U6 as an internal (RT primer sequence: 5′-CGCTT CACGA ATTTG CGTGT CAT-3′).

#### 2.2.5. Protein Extraction and Western Blot

The H9c2 or primary cardiomyocytes were grown in 6-well plates, divided into the four groups as described above, and treated for 24 h. Subsequently, the cells were collected for protein extraction and lysed in RIPA buffer (1% NP-40, 1 mmol/l Na_3_VO_4_, 1 mmol/l NaF, and 0.5 mmol/l PMSF) on ice for 30 min. The lysate was clarified by centrifugation, and the supernatant was removed. The protein concentrations were assessed by using the BCA Protein Assay Kit (Pierce), and the absorbance was read at 490 nm by means of ELISA reader. Cell lysate containing 30 *μ*g of total protein was separated by 10% SDS-PAGE and electrophoretically transferred to polyvinylidene difluoride membranes. The membranes were blocked through incubation in 5% blotting grade milk (Bio-Rad) in TBS-T (0.1% Tween 20 in TBS) and then probed overnight with the following primary antibodies: anti-STAT-phospho Y705 (Abcam), Bcl-2 (CST), Bax (CST), and *β*-actin at 4°C. Subsequently, the membrane was incubated with HRP-conjugated secondary antibodies (CST). The fluorescence signals were visualized by using SuperSignal West Pico Chemiluminescent Substrate (Pierce).

#### 2.2.6. Transfection and Dual-Luciferase Reporter Assay

Rno-miR-181a inhibitor and rno-miR negative control were synthesized by GenePharma (Shanghai). Twenty-four hours prior to transfection, H9c2 cells were plated in 6-well plates (2.5 × 10^5^ cells/well) and then transfected with miR-181a inhibitor (50 nM) or miR negative control (50 nM) by using Lipo2000 (Invitrogen). A Bcl-2-3′UTR luciferase reporter was created. The site-directed mutagenesis of the miR-181a target site was conducted by using Invitrogen. The constructs were sequenced and named pGL3-luc-Bcl-2-3′-UTR-wt or pGL3-luc-Bcl-2-3′-UTR-mut. For the reporter assays, H9c2 cells (6 × 10^4^ cells/well) were incubated in 24-well plates and cotransfected with miR-181a mimic or control mimic and pGL3-luc-Bcl-2-3′-UTR-wt or pGL3-luc-Bcl-2-3′-UTR-mut containing a firefly luciferase reporter gene by using Lipofectamine® 2000 (Invitrogen). The luciferase activity was measured for 48 h after transfection by using the Dual-Luciferase Reporter Assay System (Promega) and normalized to *Renilla* luciferase activity.

#### 2.2.7. Establishment of Animal Model

The body weights of F344 rats at the start of the experiment were 210 ± 15 g. The forty rats were randomly assigned into four groups of ten animals: (1) control group: injection of the same volume of saline at the same time; (2) prop group: 50 mg/kg propofol; (3) ADM group: 0.8 mg/kg ADM; and (4) ADM + prop group: 50 mg/kg propofol plus 0.8 mg/kg ADM. The administration was conducted by intraperitoneal injection every 3 weeks, for 6 weeks. The rats in each group were observed for psychological status, appetite, and activity, and the number of the surviving animals was recorded, and the survival rate was calculated. The body weights of the animals were weighed weekly. At the 3rd day after the last dose, the animals were sacrificed.

#### 2.2.8. TUNEL Assay

Cardiomyocyte apoptosis was detected by the TUNEL assay: the paraffin blocks were serially sectioned, routinely dewaxed, antigen repaired at 95°C for 10 min, soaked in 3% hydrogen peroxide solution for 20 min, and digested in 20 *μ*g/ml proteinase K for 30 minutes. TUNEL buffer (30 mmol/l) was prepared, stained with hematoxylin for 5 min, dipped in alcohol containing 1% hydrochloric acid for 5–10 s, washed in tap water for 25 min, and stained with 0.5% eosin for 2 min. The slides were dehydrated in gradient alcohol, cleared, and mounted. For each slide, 5 myocardial fields were randomly selected, and the numbers of TUNEL-positive cells and total cells were counted for each high magnification field, the apoptotic index (AI) was calculated, and the average AI was calculated for five fields. AI (%) = number of apoptotic cells/total cells × 100%.

#### 2.2.9. Statistics

The measurement data were presented as the mean ± S.D. and analyzed with statistical methods, including Student's *t*-test and two-way ANOVA. The statistical analysis was conducted by using the SPSS 17.0 software. The significance level (*α*) was 0.05.

## 3. Results

### 3.1. Propofol Reduced the Inhibitory Effect of ADM on Cardiomyocytes

**Figure 1:**
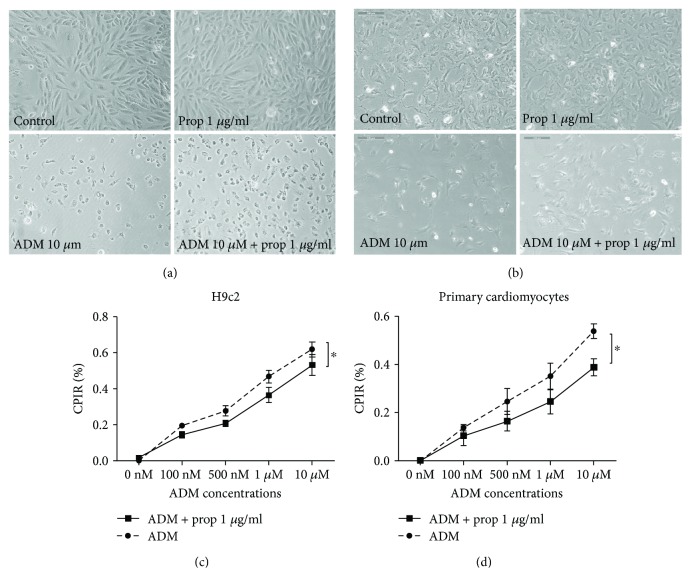
Antiproliferative effect ADM alone or combination treatment with propofol in cardiomyocytes. Morphological changes of H9c2 cells (a) and primary cardiomyocytes (b) in different treatment (control, prop 1 *μ*g/ml, ADM 10 *μ*M, and prop 1 *μ*g/ml + ADM 10 *μ*M) by microscope. Compared with the group treated with the combination of ADM with propofol, the antiproliferative effect of ADM alone on cardiomyocytes was significantly greater in H9c2 cells (*P* = 0.0253) (c) and primary cardiomyocytes (*P* = 0.0182) (d). The inhibition concentration of propofol in the combination treatment was maintained at 1 *μ*g/ml, while the concentrations of ADM were 100 nM, 500 nM, 1 *μ*M, and 10 *μ*M. Each column or data point represents the mean ± S.D. (*n* = 3). The statistical analysis was performed with two-way ANOVA using Tukey's test for pairwise comparisons. NS: not significant; ^∗^*P* ≤ 0.05 compared with the ADM group.

The *in vitro* effect of propofol, ADM, and the combinational treatment on cell growth was determined by using an MTT assay. In H9c2 cells, the antiproliferative effect of ADM alone was significantly greater (*P* = 0.0253) than the combined effect of AMD and propofol (Figure 1(c)). A similar protective effect of propofol on cardiomyocyte viability was observed in primary cardiomyocytes (*P* = 0.0182) (Figure 1(d)), which showed that propofol reduced the inhibitory effect of ADM on cardiomyocytes.

### 3.2. Propofol Suppressed the Percentage of Apoptosis Cells in ADM Treatment

**Figure 2:**
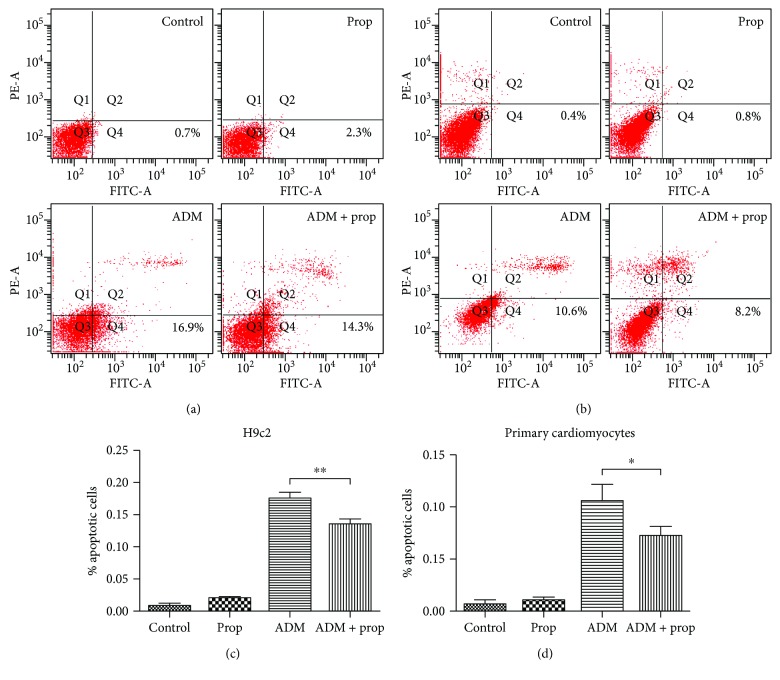
Effect of the combination of propofol and ADM induction of early apoptosis in cardiomyocytes by flow cytometry. After exposure to the different treatments for 24 h, the proportion of early apoptotic cells was analyzed using Annexin V-FITC and PI in H9c2 cells (a) and primary cardiomyocytes (b). Compared with the Adriamycin-alone group, combination treatment induced a decrease in the population of cells undergoing early apoptosis in H9c2 cells (*P* = 0.0037) (c). The phenomenon was also taken place in primary cardiomyocytes (*P* = 0.0272) (d). Each column represents the mean ± S.D. (*n* = 3). The statistical analysis was performed with Student's *t*-test. NS: not significant; ^∗^*P* ≤ 0.05 and ^∗∗^*P* < 0.01, compared with the ADM group.

To determine whether propofol preserved myocardial cell viability through intervening in the process of apoptosis, cardiomyocytes of all four groups were assessed by flow cytometry (Figure 2). The results showed that the proportion of apoptotic cells was significantly elevated to 17.6% ± 0.89% in ADM-treated cells compared with 0.87% ± 0.37% in the controls (*P* < 0.001) but was stably maintained at 13.6% ± 0.70% upon propofol treatment. There was a significantly greater decrease in apoptosis in the combination treatment group compared to ADM group in H9c2 (*P* = 0.0037) (Figure 2(c)). Similar results were also seen, with 10.6% ± 0.32% apoptosis in the ADM-alone group and 7.2% ± 0.32% in the combination group, which indicated a decrease in the population of cells undergoing early apoptosis in primary cardiomyocytes (*P* = 0.0272) (Figure 2(d)). The phenomenon indicated that propofol could reduce anthracycline-induced apoptosis in cardiomyocytes *in vitro*.

### 3.3. Propofol Reduced the Upregulation of miR-181a by ADM

**Figure 3:**
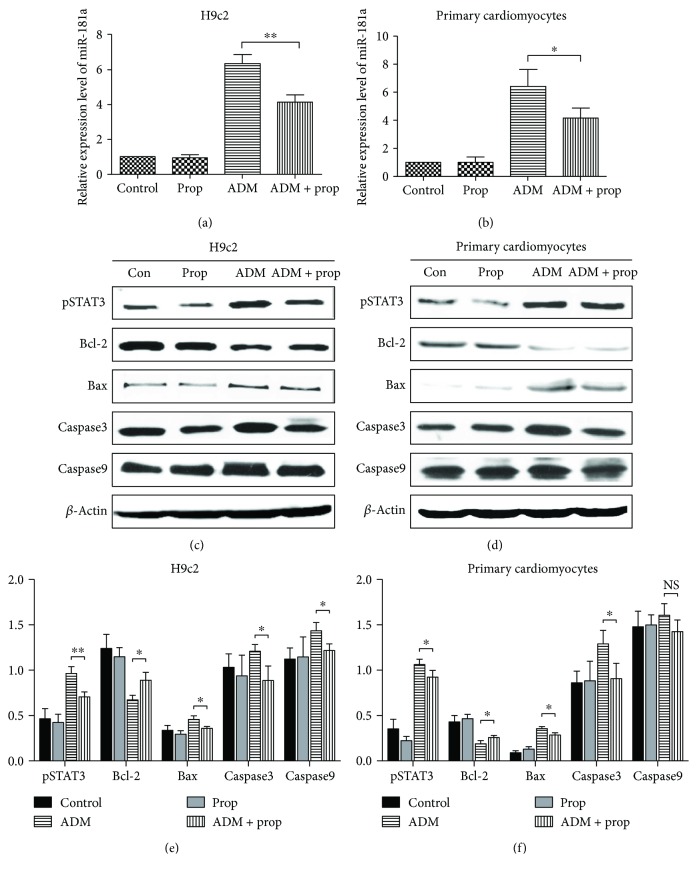
Propofol reduced the upregulation of miR181a induced by ADM and inhibited ADM-induced STAT3 activating. The expression of miR-181a in different treatment on H9c2 (a) or primary cardiomyocytes (b) tested by RT-PCR. The combination treatment upregulation of miR-181a was reduced compared to the ADM-alone group. (c-d) Phospho-STAT3, Bcl-2, Bax, Caspase3, and Caspase9 protein levels were measured in different treatment in cardiomyocytes by Western blotting. (e-f) Semiquantitative data from densitometric analysis of phospho-STAT3, Bcl-2, Bax, Caspase3, and Caspase9 are presented as relative ratio of each protein to *β*-actin. Data are expressed as mean ± SD (*n* = 3). The statistical analysis was performed with Student's *t*-test. NS: not significant; ^∗^*P* ≤ 0.05 and ^∗∗^*P* < 0.01, compared with the ADM group.

To ascertain the role of miR-181a in ADM-induced cardiomyocyte apoptosis, miR-181a expression was tested by RT-PCR. A significant upregulation of miR-181a expression was found in both ADM-alone group and the combination group (Figures 3(a) and 3(b)). The upregulation of miR-181a by the combination treatment was reduced compared with ADM alone in H9c2 (*P* = 0.0048, Figure 3(a)). Compared with the ADM group, a significant decrease in the level of miR-181a in primary cardiomyocytes was observed after the combination treatment (*P* = 0.0442, Figure 3(b)). All these data indicated that propofol could reduce the upregulation of miR-181a induced by ADM.

To explore the effect of propofol on the STAT3 pathway and apoptosis-related proteins in cardiomyocytes, the change in protein expression of phospho-STAT3, Bcl-2, Bax, Caspase3, and Caspase9 after the different treatments was detected by Western blot (Figures 3(c) and 3(d)). In H9c2, compared with control group, the phospho-STAT3 was significantly overexpressed in the ADM group. The expression of phospho-STAT3 in the combination treatment group was significantly reduced compared with that in the ADM group (*P* < 0.01), which showed that propofol inhibited the anthracycline-induced activation of STAT3; the decrease in the expression of Bax, Caspase3, and Caspase9 and increase in Bcl-2 expression were statistically significant compared with that in the ADM group (*P* < 0.05) (Figure 3(e)). Although there was no statistical difference between ADM group and combination treatment group in primary cardiomyocytes, the differential expressions of other proteins in different groups were significant (Figures 3(d) and 3(f)).

### 3.4. Propofol Reduced ADM-Induced Apoptosis by miR-181a In Vitro

**Figure 4:**
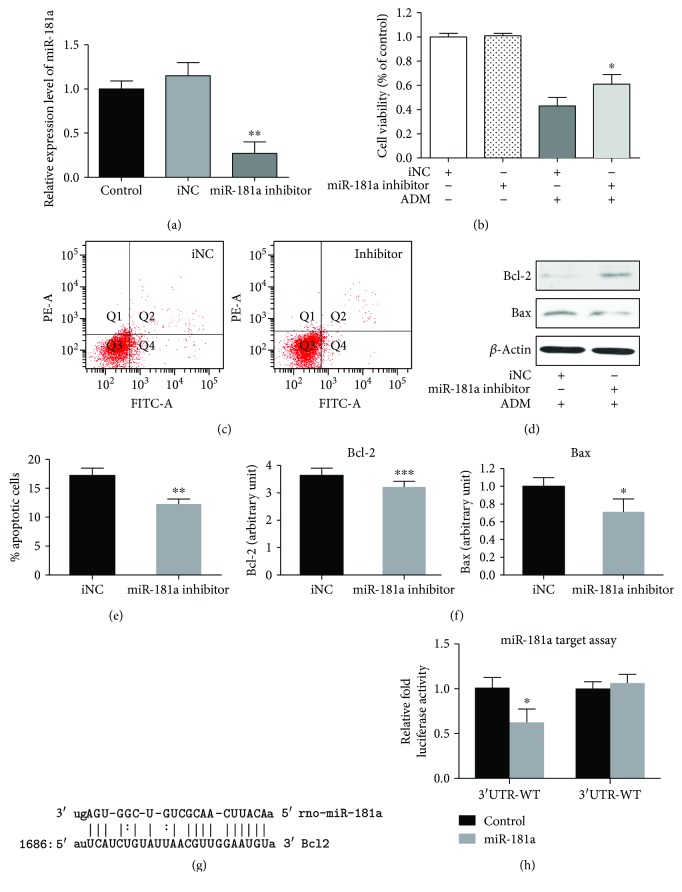
miR-181a contributes to reducing ADM-induced cardiomyocyte apoptosis by repressing Bcl-2. (a) RNAs isolated from H9c2 cells transfected with miR-181a inhibitor or control were analyzed by RT-qPCR to access the levels of miR-181a. The expression of miR-181a was reduced by miR-181a inhibitor transfection. (b) The cell viability of H9c2 cells transfected with miR-181a inhibitor or control in different treatment was tested by MTT assay. (c-e) After 24 h exposure of ADM treatment, the proportion of apoptotic cells was decreased in miR-181a inhibitor cells compared with control cells (*P* = 0.006). (d-f) The silencing of miR-181a in H9c2 cell treatment with ADM resulted in increased Bcl-2 (*P* = 0.0007) and decreased Bax (*P* = 0.0495) protein levels. (g) Rat Bcl-2 has strong rno-miR-181a-binding sites at its 3′-UTR. MiR-181a seed pairing in the target regions is shown as vertical lines. (h) Relative luciferase activity was analyzed after wild-type or mutant Bcl-2-3′UTR reporter plasmids were cotransfected with miR-181a mimic in H9c2 cells as shown. Compared to the negative control, miR-181a repressed the activity of luciferase fused to the WT Bcl-2-3′UTR (*P* = 0.0299), while it failed to repress the mutated one (*P* = 0.4611). Data are representative of three independent experiments. Data from triplicate experiments were showed as mean ± SD (*n* = 3). The statistical analysis was performed with Student's *t*-test. ^∗^*P* ≤ 0.05; ^∗∗^*P* < 0.01; ^∗∗∗^*P* < 0.001.

As shown previously, propofol played a protective role in ADM-induced apoptosis, whereas the expression of miR-181a was significantly reduced in the ADM + prop group compared with that in the ADM group. To confirm the critical role of miR-181a in ADM-induced cardiomyocyte apoptosis in H9c2 cells, we transfected rno-miR-181a inhibitor and rno-miR-181a control into H9c2 cells and detected changes in apoptosis. The qRT-PCR assays showed that the expression of miRNA-181a in H9c2 cells transfected with the miRNA-181a inhibitor plasmid was significantly lower than that of H9c2 cells transfected with a miRNA-181a NC plasmid (*P* = 0.0015, Figure 4(a)). The viability of cardiomyocytes with different miRNA-181a levels was detected by an MTT assay (Figure 4(b)). Compared with the iNC + ADM group, the cell viability of myocardial cells transfected with the miRNA-181a inhibitor was significantly increased (*P* = 0.0427) in miR-181a-inhibitor + ADM group, which indicated that the inhibition of miR-181a in cardiomyocytes attenuated ADM-induced cardiomyocyte death. Compared with the iNC group (17.2% ± 1.3%), a significant decrease (*P* = 0.006) in the early apoptosis rate of miR-181a inhibitor cells (12.3% ± 0.9%) was observed (Figures 4(c) and 4(e)). The differential expression of Bcl-2 and Bax after ADM treatment in miR-181a-depleted cells and control cells was examined by Western blotting (Figure 4(f)). Compared with the control cells, the expression of Bcl-2 was significantly increased (*P* = 0.0007) and the expression of Bax was significantly decreased (*P* = 0.048) in miR-181a-depleted cells.

To delineate the underlying mechanism of miR-181a in the protective effect of ADM-induced cardiomyocyte apoptosis, we searched for potential target genes of miR-181a by using several bioinformatics tools, such as TargetScan, miRanda, and PicTar. The computational analysis revealed that there was a binding site on the 3′ UTR of Bcl-2 for miR-181a, which was highly conserved among different species (Figure 4(g)). To ascertain whether these miR-181a-binding sequences directly contributed to the negative regulation of Bcl-2 expression, we used a luciferase assay to verify that Bcl-2 was the direct target gene that miR-181a regulates H9c2 cells. We cloned the Bcl-2-3′UTR region into the pGL3-control plasmid containing the luciferase reporter gene. The plasmids containing pGL3-Bcl-2-wt or pGL3-Bcl-2-mut and the miR-181a mimic or control mimic were cotransfected. If miR-181a was capable of binding to the 3′UTR region of the Bcl-2 gene, the luciferase activity decreased in the cells transfected with pGL3-Bcl-2-wt plasmid as the expression of miR-181a increased. As shown in Figure 4(h), luciferase activity was decreased significantly by transfection of the miR-181a mimic when the wild-type 3′UTR of Bcl-2 was present (*P* = 0.0299), whereas no difference was found after the transfection of the pGL3-Bcl-2-mut vector (*P* = 0.4611). Therefore, miR-181a suppressed Bcl-2 expression through the direct targeting of the Bcl-2 transcript in H9c2.

### 3.5. The Protective Effect of Propofol on Rat Cardiomyocytes In Vivo

**Figure 5:**
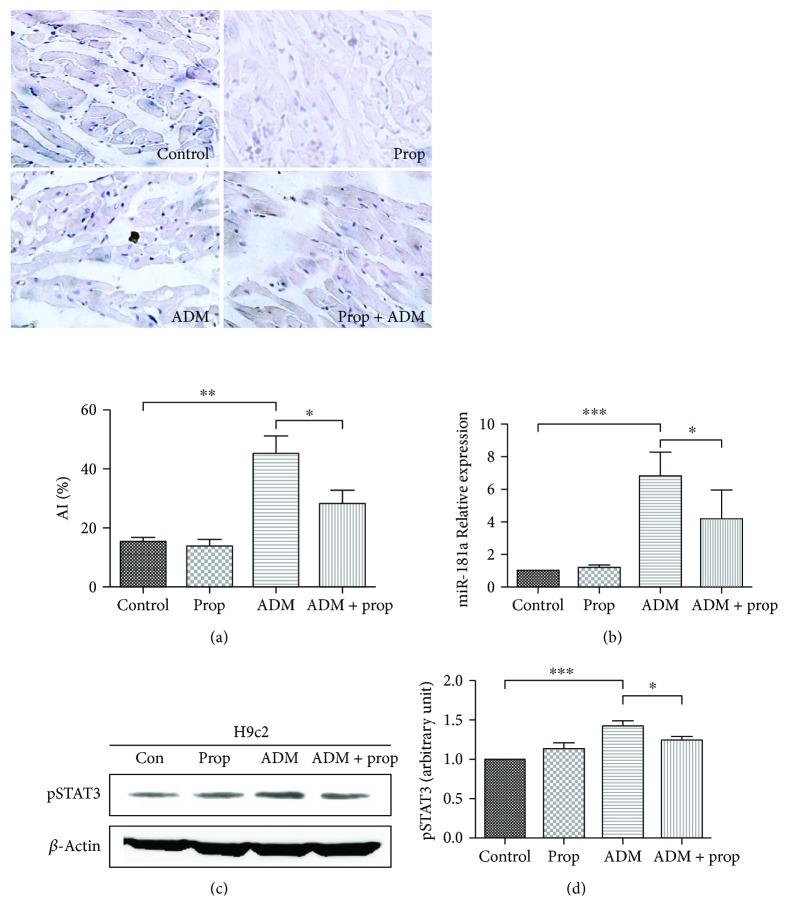
Propofol could reduce the apoptosis cells induced by ADM in cardiomyocytes *in vivo.* (a) Myocardial sections of different treatments were stained for TUNEL assay to measure apoptosis index (AI). The AI of rat heart tissue was significantly decreased in ADM + prop group compared with ADM-alone group (*P* = 0.0172). (b–d) RNAs or proteins isolated from heart tissues in different group were analyzed by RT-qPCR or Western blot to access the levels of miR-181a (b) or phospf-STAT3 (c-d). Both miR-181a and phospho-STAT3 were overexpressed in ADM group compared to ADM + prop group. The statistical analysis was performed with Student's *t*-test. NS: not significant; ^∗^*P* ≤ 0.05, ^∗∗^*P* ≤ 0.01 and ^∗∗∗^*P* < 0.001 compared with the control group.

F344 rats were randomly divided into the following groups: (1) control group; (2) prop group; (3) ADM group; (4) ADM + prop group. The apoptosis index in rat heart tissue after different treatments was determined by TUNEL staining, and the expression of miR-181a and phospho-STAT3 was determined by RT-qPCR or Western blotting (Figure 5). The TUNEL study showed similar results in vitro, with a low number of TUNEL-positive nuclei in the specimens of the prop group and a prevalence of TUNEL-positive nuclei in the ADM group. Compared with ADM alone, the apoptosis index of rat heart tissue was significantly increased in the ADM + prop group (*P* = 0.0172). A significant reduction in the expression of miR-181a and phospho-STAT3 occurred in the ADM + prop group compared with that in the ADM-only group, as shown in Figures 5(b) and 5(d) (*P* < 0.05). This indicated that anthracycline-induced cardiomyocyte apoptosis and increased the expression of miR-181a and phospho-STAT3 *in vivo.*

## 4. Discussion

The potential significance of microRNAs in myocardial injury has been highlighted by recent mechanistic research [17]. The results of this study revealed that propofol provided cardioprotective effects against ANT-induced cardiotoxicity, which were mediated by miR-181a both *in vitro* and *in vivo*.

In previous studies, ANT-induced cardiotoxicity was shown to involve cardiomyocyte apoptosis [18]. It has been generally recognized that the main target of ANT antitumor action was topoisomerase II*α* (TOP2*α*), through the formation of the Top2-ANT-DNA complex, which causes double-stranded DNA breaks [19–21], which are able to trigger the apoptosis of cancer cells, apparently via the p53-dependent pathway [22]. In this study, we found that anthracycline could induce cardiomyocyte apoptosis and upregulated the expression of miR-181a *in vitro* and *in vivo*, which revealed that miR-181a was related to anthracycline-induced cell apoptosis.

Propofol, an anesthetic with pluripotent cytoprotective properties against various toxic insults, was shown to ameliorate ischemia-reperfusion injury in multiple organs such as the heart [12, 23], brain [24], and kidney [25]. The previously established cardioprotective actions of propofol on ANT-induced cardiotoxicity include the reduction of oxidative damage [26] and the inhibition or attenuation of cardiomyocyte apoptosis by p53/PKC-*δ* [26] and the PI3K/AKT pathway [16]. However, the cardioprotective mechanism of propofol that counteracts doxorubicin-induced cellular apoptosis is currently unclear. Our data confirmed that the viability of cardiomyocytes in the group treated with the combination of ADM and propofol was significantly higher than those in the ADM-alone group (*P* < 0.05) (Figures 1(c) and 1(d)). Compared with the ADM groups, a significantly greater decrease in apoptosis was observed in the combination treatment group, which indicated that propofol reduced cell apoptosis by anthracycline in cardiomyocytes *in vitro* (Figures 2(a) and 2(b)) and *in vivo* (Figure 5(a)). Similar protective effects of propofol on ANT-induced apoptosis in an *in vitro* model were also described by both Lai et al. [26] and Sun et al. [16].

miR-181a, widely present in human organs, has been reported to modulate cell proliferation, migration, apoptosis, and tumorigenesis [27–29]. A low global expression of miR-181a was observed in breast and colon cancer cells [30]. In contrast, miR-181a was upregulated in patients with AML who had a favorable outcome [31]. Therefore, it was undetermined whether miR-181a functioned as oncogene or tumor suppressor. In the present study, the depressed level of miR-181a was mostly restored through the addition of propofol to anthracycline-treated cardiomyocytes *in vitro* (Figures 2(a) and 2(b)) and *in vivo* (Figure 5(b)), which indicated that miR-181a was involved in the propofol-mediated relief of doxorubicin-induced cardiomyopathy. The activity of transcription factor STAT3, which orchestrates the induction of miR-181a [32], was lower in the ADM + prop group than in the ADM-alone group (Figures 3(c) and 5(c)), which reflected the significance of miR-181a in the cardioprotective mechanism of propofol. The correlation of miR-181a and anthracycline resistance in patients with triple-negative breast cancer was reported by Ouyang et al. [33]. In a recent study by Zhu et al., miR-181a modulated cardiomyocyte apoptosis induced by Necrotic-S-treated dendritic cells (DCs) in hypoxic conditions [8]. Our study showed that propofol could attenuate cardiomyocyte apoptosis through reducing the activation of apoptosis-related protein Bax, Caspase3, and Caspase9, while upregulating the expression of Bcl-2 (Figures 3(d)–3(f)). Cellular protection of propofol in cardiomyocytes against hypoxia/reoxygenation injury with high-glucose exposure had been provided in Deng et al. [12] study though reducing the cell apoptosis evidenced by decreased Bax/Bcl-2 ratio and decreased Caspase3 and Caspase9 expression.

In addition, we demonstrated that miR-181a suppressed Bcl-2 expression by directly targeting of the Bcl-2 transcript in H9c2 cells (Figure 4(h)). Some researchers demonstrated that miR-181a regulated the expression of the Bcl-2 family in different cell types [32, 34]. Bcl-2, for which the gene product is known to have a role in apoptosis reduction, was one of the direct target genes of miR-181a [35] and inhibited mitochondrial metabolism and ADM-induced apoptosis in cancer cells [36]. However, this study was the first to confirm the direct regulation between miR-181a and Bcl-2 in rat cardiomyocytes. Wang et al. studied the target genes of miR-181a in cardiomyocytes and concluded that miR-181a regulated the expression of Gpx1 and that Gpx1 directly influenced the expression of Bcl-2 and played an important role in the regulation of the mitochondrial apoptotic pathway in cardiomyocytes challenged by oxidative stress [9].

In summary, we found that propofol reduced apoptosis induced by ANT in cardiomyocytes *in vitro* and *in vivo*. The protective effect may be related to the regulation of the miR-181a/Bcl-2 pathway and the observed negative correlation between miR-181a and Bcl-2 in cardiomyocytes. In future studies, we aim to confirm if propofol regulates cardiomyocyte apoptosis through the STAT3/miR-181a/Bcl-2 pathway and determine the relationship between STAT3 and miR-181a in cardiomyocytes.

## References

[1] Minotti G., Menna P., Salvatorelli E., Cairo G., Gianni L. (2004). Anthracyclines: molecular advances and pharmacologic developments in antitumor activity and cardiotoxicity. *Pharmacological Reviews*.

[2] Vedam K., Nishijima Y., Druhan L. J. (2010). Role of heat shock factor-1 activation in the doxorubicin-induced heart failure in mice. *American Journal of Physiology Heart and Circulatory Physiology*.

[3] Su H., Gorodny N., Gomez L. F. (2015). Noninvasive molecular imaging of apoptosis in a mouse model of anthracycline-induced cardiotoxicity. *Circulation: Cardiovascular Imaging*.

[4] Zhang S., Meng T., Liu J., Zhang X., Zhang J. (2015). Cardiac protective effects of dexrazoxane on animal cardiotoxicity model induced by anthracycline combined with trastuzumab is associated with upregulation of calpain-2. *Medicine*.

[5] Tang Y., Wang Y., Park K. M. (2015). MicroRNA-150 protects the mouse heart from ischaemic injury by regulating cell death. *Cardiovascular Research*.

[6] van Rooij E. (2011). The art of microRNA research. *Circulation Research*.

[7] Small E. M., Olson E. N. (2011). Pervasive roles of microRNAs in cardiovascular biology. *Nature*.

[8] Zhu J., Yao K., Guo J. (2017). miR-181a and miR-150 regulate dendritic cell immune inflammatory responses and cardiomyocyte apoptosis via targeting JAK1-STAT1/c-Fos pathway. *Journal of Cellular and Molecular Medicine*.

[9] Wang L., Huang H., Fan Y. (2014). Effects of downregulation of microRNA-181a on H_2_O_2_-induced H9c2 cell apoptosis via the mitochondrial apoptotic pathway. *Oxidative Medicine and Cellular Longevity*.

[10] Chidambaran V., Costandi A., D'Mello A. (2015). Propofol: a review of its role in pediatric anesthesia and sedation. *CNS Drugs*.

[11] Zhao D., Li Q., Huang Q. (2015). Cardioprotective effect of propofol against oxygen glucose deprivation and reperfusion injury in H9c2 cells. *Oxidative Medicine and Cellular Longevity*.

[12] Deng F., Wang S., Zhang L. (2018). Propofol through upregulating caveolin-3 attenuates post-hypoxic mitochondrial damage and cell death in h9c2 cardiomyocytes during hyperglycemia. *Cellular Physiology and Biochemistry*.

[13] Li H., Tan J., Zou Z., Huang C. G., Shi X. Y. (2011). Propofol post-conditioning protects against cardiomyocyte apoptosis in hypoxia/reoxygenation injury by suppressing nuclear factor-kappa B translocation via extracellular signal-regulated kinase mitogen-activated protein kinase pathway. *European Journal of Anaesthesiology*.

[14] Rogers C. A., Bryan A. J., Nash R. (2015). Propofol cardioplegia: a single-center, placebo-controlled, randomized controlled trial. *The Journal of Thoracic and Cardiovascular Surgery*.

[15] Ansley D. M., Raedschelders K., Choi P. T., Wang B., Cook R. C., Chen D. D. Y. (2016). Propofol cardioprotection for on-pump aortocoronary bypass surgery in patients with type 2 diabetes mellitus (PRO-TECT II): a phase 2 randomized-controlled trial. *Canadian Journal of Anaesthesia*.

[16] Sun X., Gu J., Chi M., Li M., Lei S., Wang G. (2014). Activation of PI3K-Akt through taurine is critical for propofol to protect rat cardiomyocytes from doxorubicin-induced toxicity. *Canadian Journal of Physiology and Pharmacology*.

[17] Xin Y., Yang C., Han Z. (2016). Circulating miR-499 as a potential biomarker for acute myocardial infarction. *Annals of Translational Medicine*.

[18] Arola O. J., Saraste A., Pulkki K., Kallajoki M., Parvinen M., Voipio-Pulkki L. M. (2000). Acute doxorubicin cardiotoxicity involves cardiomyocyte apoptosis. *Cancer Research*.

[19] Muindi J. R., Sinha B. K., Gianni L., Myers C. E. (1984). Hydroxyl radical production and DNA damage induced by anthracycline-iron complex. *FEBS Letters*.

[20] Capranico G., Zunino F. (1992). DNA topoisomerase-trapping antitumour drugs. *European Journal of Cancer*.

[21] Tewey K., Rowe T., Yang L., Halligan B., Liu L. (1984). Adriamycin-induced DNA damage mediated by mammalian DNA topoisomerase II. *Science*.

[22] Ruiz-Ruiz C., Robledo G., Cano E., Redondo J. M., Lopez-Rivas A. (2003). Characterization of p53-mediated up-regulation of CD95 gene expression upon genotoxic treatment in human breast tumor cells. *The Journal of Biological Chemistry*.

[23] Xia Z., Huang Z., Ansley D. M. (2006). Large-dose propofol during cardiopulmonary bypass decreases biochemical markers of myocardial injury in coronary surgery patients: a comparison with isoflurane. *Anesthesia & Analgesia*.

[24] Luo T., Wu J., Kabadi S. V. (2013). Propofol limits microglial activation after experimental brain trauma through inhibition of nicotinamide adenine dinucleotide phosphate oxidase. *Anesthesiology*.

[25] Li Y., Zhong D., Lei L., Jia Y., Zhou H., Yang B. (2015). Propofol prevents renal ischemia-reperfusion injury via inhibiting the oxidative stress pathways. *Cellular Physiology and Biochemistry*.

[26] Lai H. C., Yeh Y. C., Wang L. C. (2011). Propofol ameliorates doxorubicin-induced oxidative stress and cellular apoptosis in rat cardiomyocytes. *Toxicology and Applied Pharmacology*.

[27] Rang Z., Wang Z., Pang Q., Wang Y., Yang G., Cui F. (2016). MiR-181a targets PHLPP2 to augment AKT signaling and regulate proliferation and apoptosis in human keloid fibroblasts. *Cellular Physiology and Biochemistry*.

[28] Parikh A., Lee C., Joseph P. (2014). microRNA-181a has a critical role in ovarian cancer progression through the regulation of the epithelial-mesenchymal transition. *Nature Communications*.

[29] Liu Z., Sun F., Hong Y. (2017). MEG2 is regulated by miR-181a-5p and functions as a tumour suppressor gene to suppress the proliferation and migration of gastric cancer cells. *Molecular Cancer*.

[30] Li Y., Kuscu C., Banach A. (2015). miR-181a-5p inhibits cancer cell migration and angiogenesis via downregulation of matrix metalloproteinase-14. *Cancer Research*.

[31] Brauer-Hartmann D., Hartmann J. U., Wurm A. A. (2015). PML/RAR*α*-regulated miR-181a/b cluster targets the tumor suppressor RASSF1A in acute promyelocytic leukemia. *Cancer Research*.

[32] Niu J., Xue A., Chi Y. (2016). Induction of miRNA-181a by genotoxic treatments promotes chemotherapeutic resistance and metastasis in breast cancer. *Oncogene*.

[33] Ouyang M., Li Y., Ye S. (2014). MicroRNA profiling implies new markers of chemoresistance of triple-negative breast cancer. *PLoS One*.

[34] Ouyang Y. B., Lu Y., Yue S., Giffard R. G. (2012). miR-181 targets multiple Bcl-2 family members and influences apoptosis and mitochondrial function in astrocytes. *Mitochondrion*.

[35] Zhu Y., Wu J., Li S. (2013). The function role of miR-181a in chemosensitivity to adriamycin by targeting Bcl-2 in low-invasive breast cancer cells. *Cellular Physiology and Biochemistry*.

[36] Singh T. R., Shankar S., Chen X., Asim M., Srivastava R. K. (2003). Synergistic interactions of chemotherapeutic drugs and tumor necrosis factor-related apoptosis-inducing ligand/Apo-2 ligand on apoptosis and on regression of breast carcinoma *in vivo*. *Cancer Research*.

